# A Comparative Analysis of 2D and 3D Tasks for Virtual Reality Therapies Based on Robotic-Assisted Neurorehabilitation for Post-stroke Patients

**DOI:** 10.3389/fnagi.2016.00205

**Published:** 2016-08-26

**Authors:** Luis D. Lledó, Jorge A. Díez, Arturo Bertomeu-Motos, Santiago Ezquerro, Francisco J. Badesa, José M. Sabater-Navarro, Nicolás García-Aracil

**Affiliations:** Biomedical Neuroengineering Group, Miguel Hernández University of ElcheElche, Spain

**Keywords:** virtual reality, rehabilitation robotics, post-stroke, sensorimotor function, upper extremity

## Abstract

Post-stroke neurorehabilitation based on virtual therapies are performed completing repetitive exercises shown in visual electronic devices, whose content represents imaginary or daily life tasks. Currently, there are two ways of visualization of these task. 3D virtual environments are used to get a three dimensional space that represents the real world with a high level of detail, whose realism is determinated by the resolucion and fidelity of the objects of the task. Furthermore, 2D virtual environments are used to represent the tasks with a low degree of realism using techniques of bidimensional graphics. However, the type of visualization can influence the quality of perception of the task, affecting the patient's sensorimotor performance. The purpose of this paper was to evaluate if there were differences in patterns of kinematic movements when post-stroke patients performed a reach task viewing a virtual therapeutic game with two different type of visualization of virtual environment: 2D and 3D. Nine post-stroke patients have participated in the study receiving a virtual therapy assisted by PUPArm rehabilitation robot. Horizontal movements of the upper limb were performed to complete the aim of the tasks, which consist in reaching peripheral or perspective targets depending on the virtual environment shown. Various parameter types such as the maximum speed, reaction time, path length, or initial movement are analyzed from the data acquired objectively by the robotic device to evaluate the influence of the task visualization. At the end of the study, a usability survey was provided to each patient to analysis his/her satisfaction level. For all patients, the movement trajectories were enhanced when they completed the therapy. This fact suggests that patient's motor recovery was increased. Despite of the similarity in majority of the kinematic parameters, differences in reaction time and path length were higher using the 3D task. Regarding the success rates were very similar. In conclusion, the using of 2D environments in virtual therapy may be a more appropriate and comfortable way to perform tasks for upper limb rehabilitation of post-stroke patients, in terms of accuracy in order to effectuate optimal kinematic trajectories.

## 1. Introduction

Virtual Reality (VR) is a technology platform that allows developing computer generated environments which the subjects can explore and interact with any type of object or events to perform perspectives and motor tasks. VR gives an accurate way to control all the elements of a scene and the objectives, adjusting each task to a specific user. The main feature that the VR provides is the possibility of repeating the same task in any moment, modifying factors such as level of complexity, time and intensity of the practice. In this way, the virtual therapy may be used to promote motor learning and rehabilitation due to the VR can be adjusted to generate environment, scenario, or activity that allows for the user practice motor skills to improve the experience-dependent neural plasticity (Doyon and Benali, 2005). The possibility of modifying factors such as the repetition, intensity, time, and specificity of the activities of the virtual therapies is beneficial for this type of neural recovery (Kleim and Jones, 2008). In recent years, some scientific and clinical trials have demonstrated the effectiveness of VR as an intervention tool for the rehabilitation of different injuries with specific neurological conditions (Burdea, 2002; Crosbie et al., 2007). However, a control device to interact with virtual activities is required, depending of the limb affected by the disease. There is a wide panorama on rehabilitation systems for upper limb that use robotic technology including virtual reality visualization (Maciejasz et al., 2014). In some studies, repetitive movements guiaded by robotic devices and directed by virtual reality improve the motor control in patients with upper limb injuries (Merians et al., 2006). Beside this, there are some clinical studies about the development of VR systems to deliver rehabilitation therapies for motor recovery of hand function (Jack et al., 2001) or to improve the performance of activities of daily living in post-stroke patients (Laver et al., 2012; Turolla et al., 2013). Furthermore, a navigation environment in three dimensions (3D) has been implemented to explore the influence on aging in the episodic memory (Jebara et al., 2014). In Fluet and Deutsch (2013), an overview of virtual reality studies for sensorimotor rehabilitation post-stroke has been performed to evaluate a comparative efficacy between VR and standard of care and/or differences in VR delivery methods, using different categories.

Several studies suggest that the robotic technology can be used to improve the quality and the evaluation in the neurological rehabilitation (Garcia et al., 2011), enhancing the productivity and reducing costs in that field. Recent developments in robotic technology can help to perform a most objective and reliable analysis of the therapies that are applied to the patients with neurological injuries (Badesa et al., 2012, 2014a,c). That is because this type of devices are able to record kinematic and kinetic data. From this data, useful markers can be extracted to quantify the motor recovery process during the therapy (Volpe et al., 2009; Einav et al., 2011; Bertomeu-Motos et al., 2015; Papaleo et al., 2015). Recently in Norouzi-Gheidari et al. (2012), it is shown that the rehabilitation sessions performed with the robotic device get better recovery outcomes than the conventional therapy during the rehabilitation of the upper limb of stroke patients. For these reasons, the rehabilitation with robotic devices can provide an enhancement in the quality of patient's life, giving them most independence in the daily life activities (Pollock et al., 2014).

The use of more complex and realistic VR systems in the neurorehabilitation therapies assisted by robotic device is increasing. The combination of robotic systems for neuromotor rehabilitation and virtual reality takes advantages of both techniques such as: to increase the patient's motivation; to enhance the variability and adaptability; transparent storage of the data provided by the robotic system and the VR system separately; online recording of the data for remote verification; possibility to replicate any environment of the daily life without having the physical. With this methodology, a more effective therapeutic treatment and a better recovery of the patient is accomplished (González et al., 2015).

There are a two important issues concerning the virtual reality: one is related to how the virtual environment may be perceived by the user using different visualization platforms, and the other one is related to graphic content. Regarding the first appointment, different visualization platforms exist such as computer monitors, head-mounted-displays (HMDs) or large screen-projection-systems (SPS). Each platform has a particular way to apply the virtual therapies taking into account therapeutic goals and may provide different benefits that are suitable for the patient's needs. In Rand et al. (2005), the effects of viewing the same virtual environment through a HMD (3D platform) and a computer monitor (2D platform) have been compared in young and older subjects. Conversely, a 3D virtual enviroment shown through a HMD and a SPS (2D platform) have been analyzed by Subramanian and Levin (2011), evaluating the motor performance with respect to the kinematic movements in healthy and post-stroke subjects. In both studies, better outcomes have been obtained when the virtual environment was shown in the 2D platform visualization, in a computer monitor and a SPS respectively. However, this studies have focused in the visualization platform and the same environments have been presented respectively in the experiments without taking account the type of graphic content that are shown (2D or 3D graphics).

Regarding the second issue appointed above about the graphic content, there are studies about VR systems with environments based on 2D graphics and others in 3D graphics. In García-Betances et al. (2015) an overview of recent VR technology for Alzheimer's disease applications has performed, and these systems use conventional 2D graphics display or 3D graphics indistinctly. Similarly occurs with the brain damage rehabilitation in Rose et al. (2005), post-stroke studies such as Merians et al. (2006), Saposnik (2016), Henderson et al. (2007), Mottura et al. (2015). Therefore, there is a wide panorama on virtual rehabilitation in the scientific literature. However, an objective comparison about how affects the visualization of 2D graphics display and 3D virtual environment to the motion perception in post-stroke subjects have not been addressed yet. That means, there is no evidence that shows if it is better or not to perform virtual rehabilitation tasks produced by 2D or 3D graphics. The visual perception of the virtual objects can be incremented using 3D graphics, in such a way that tasks based in the daily life designs are more similar to the reality. While a 2D graphics allow a more simple representation of the tasks. The two perspectives must be tested to evaluate what kind of visual representation provides better quality of motor performance in terms of movement kinematics. This evaluation can be carried out when the subject performs the same movement to complete the targets in both types of visualization. Therefore, the robotic devices can be used to restrict this movement and extract objectively quantitative data. This way, the neuro-rehabilitation therapies can be adapted to each patient (Morales et al., 2014; Lledó et al., 2015a).

In this study, the effects of applying therapeutic games in two or three dimensions in the virtual therapies assisted by a robotic device are evaluated and their outcomes are compared. In this way, quantitative data is provided to evaluate the influence of the virtual therapy and to asses what kind of virtual environment is adjusted better to each patient in terms of usability, confidence, and comfort. Therefore, the main objective of this study was to determine if there are differences in the movement kinematics parameters recorded by the robotic device that assess the patient's motor performance in 2D and 3D virtual tasks. To do this, two visual tasks have been designed modifying the immersion level using graphics in two and three dimensions, but the kinematic target of the two visual tasks was remained.

## 2. Materials and methods

### 2.1. Patients

The study has been performed in a hospital of attention to chronic patients and long-stay. The experiment protocol of the proposal study was approved by the Medical Ethics Committee. The medical team has been responsible for including patients who are receiving physiotherapy and occupational therapy treatment. All patients have been informed properly by the medical staff and they gave written consent before starting the study, indicating that they understood the purpose and requirements of the study.

The inclusion criteria were: adults with hemiparesis/hemiplegia secondary to stroke in subacute phase (between 1 and 6 months after the injury). The criteria with respect of the muscular conditions of the upper-limb were (i)muscular tone with punctuation below 2 in the Modified Ashwoth Scale (Bohannon and Smith, 1987), (ii)muscular balance in shoulder abduction and elbow flexion on the basis of the Motor Index ≥ 2 (Collin and Wade, 1990). In the selection process, the inclusion of patients with the following injures was avoided: painful shoulder, apraxia, uncontrolled trunk in seating system, diagnostics with hand effects (as arthritis or other rheumatologic diseases), severe perceptual deficits, stroke of posterior circulation (vertebrobasilar system), linguistic deficits that prevent useful communication. The patients have to be oriented to the three spheres (social, temporal, and spatial), with capacity of collaboration and understanding the tasks instructions and all relevant information from the study. After selection process, nine post-stroke patients (age 40–70 years of both genders) have taken part in the study. The pathologies diagnosed are varied such as pontine and artery cerebral middle infarction, ischemic myelitis, glioblastoma hematoma, parietal and basal ganglia hemorrhage, with a type of diagnosys ischemic, hemorrhagic or myelopathy and left or right laterality. The main characteristics of the study participants and their clinical diagnostic are shown in Table 1.

**Table 1:** **Clinical characteristics of the study participants**.

**Patient**	**Sex**	**Age (years)**	**Diagnosis**	**Diagnostic type**	**Location**	**Laterality**
1	Female	69	Ischemic myelitis	Myelopathy	Medullary	Tetraparesia
2	Male	40	GGBB hemorrhage	Hemorrhagic	Basal ganglia	Right
3	Male	41	GB Hematoma	Hemorrhagic	Basal ganglia	Left
4	Male	46	Undetermined ACM infarct	Ischemic	Parietal	Left
5	Female	66	Pontine infarction	Ischemic	Brainstem	Left
6	Female	41	Parietal hemorrhage	Hemorrhagic	Parietal	Right
7	Male	53	Undetermined ACM stroke	Ischemic	Parietal	Right
8	Female	41	Cerebral hemorrhage	Hemorrhagic	Frontal	Left
9	Female	46	Cerebral hemorrhage	Hemorrhagic	Frontal	Left

ACM, Artery Cerebral Middle; GB, Glioblastoma; GGBB, Basal Ganglia.

### 2.2. Neurorehabilitation system

**Figure 1:**
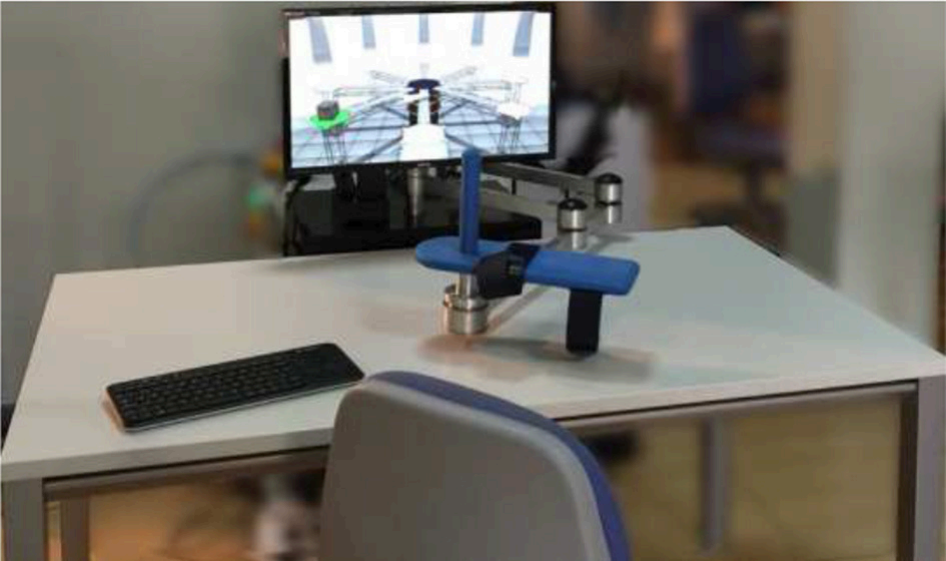
**Neurorehabilitation system based in PUPArm robot**.

The neurorehabilitation system used to perform the motor therapy and get all the objective information about the proposed study is formed by a PUPArm robot system (Badesa et al., 2014c) and a visualization subsystem. This system was designed and developed by Biomedical Neuroengineering Group at Miguel Hernandez University of Elche as a rehabilitation robot for patients with stroke or other neurological disorders. The neurorehabilitation system is shown in Figure 1.

The robotic mechanism consists of four metallic bars, similar to the MIT-MANUS rehabilitation robot (Krebs et al., 1998). These bars are connected as a parallelogram and are driven by pneumatic swivel modules. This structure provides a planar two-dimensional manipulator with two degrees of freedom. The manipulator remains fixed to a table. Consequently, the horizontal movement to upper limb of the subjects is permitted by the system, involving flexion and extension of the elbow and shoulder, and horizontal abduction and adduction. On the other hand, the visualization subsystem is composed of a monitor computer with a custom developed software called REVIRE which is used as VR simulation system to display activities in coordination with the robot's movements. A computer is responsible to coordinate in time-real the pneumatic actuators, the targets of the tasks and the feedback to the user. The system is capable to record information about the patient's progress in rehabilitation, based in parameters such as position, velocity and forces. All data is registered by robot sensors. These data are processed to provide an objective assessment to the therapist.

### 2.3. Virtual tasks

**Figure 2:**
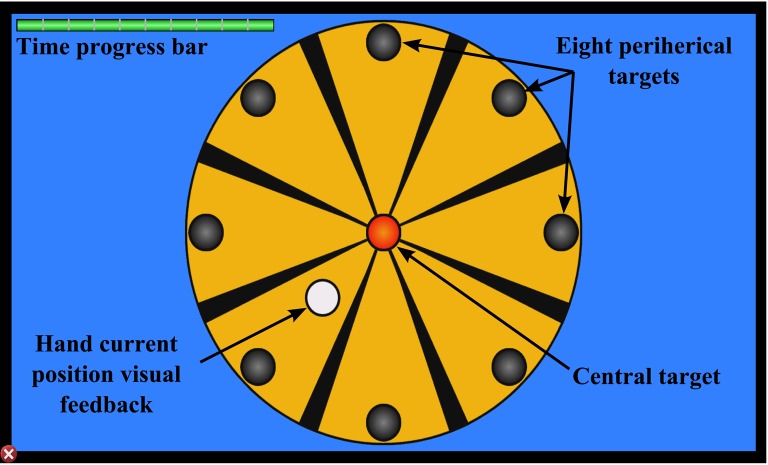
**Targets and visual feedback of the 2D task**.

The virtual task with the 2D environment consists on a roulette formed by a central target and eight peripheral targets. These targets were circles with a 1 cm of radius. The eight peripheral targets were distributed uniformly on the circumference of the circle and placed 10 cm from the center target. The main purpose of this task was to reach one of the eight peripheral targets from central target by controlling the robot end-effector attached to the subject's hand. The next target remain illuminated. To do that, the therapy task is displayed on the monitor of the neurorehabilitation system with a visual-guided reinforcement represented by a white circle of 1cm of radius which indicate the current position of the robot end-effector. A screenshot with the 2D environment of the virtual task and its structural information is shown in Figure 2. This virtual task and the neurorehabilitation system have been used in our previous studies about the age influence in the sensorimotor function of the upper limb (LLinares et al., 2013) or special cases of neurological disorder (Badesa et al., 2014b).

**Figure 3:**
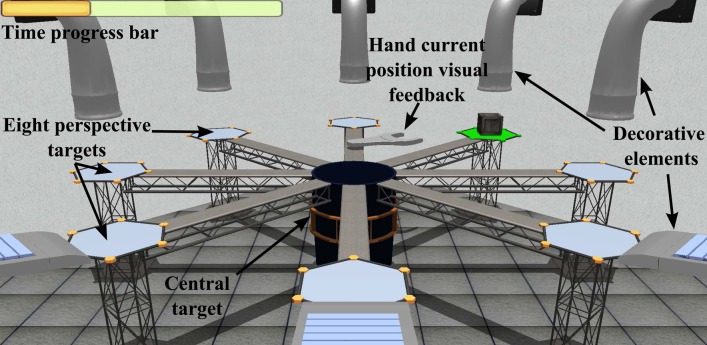
**Targets and visual feedback of the 3D task**.

To complete this comparative study, a 3D virtual task has been designed following the same target criteria used in the 2D roulette in order to perform the same type of movements. The 3D virtual task has been developed using the implementation pattern of virtual simulators explained in Lledó et al. (2015b). Ogre3D (Steve, 2013) is used as engine of graphical rendering and the physic engine NVIDIA PhysX Nvidia (2011) applies an extra degree of realism to the collision between elements of the 3D environment, whose graphical meshes are designed with the modeling tool called Blender. The environment of the 3D virtual task simulates a box factory with perspective viewing where the scene converges to the central point of the screen. The graphical scenario consists of eight platforms and a central deposit, that are equivalent to the eight peripheral targets and the center target of the 2D roulette. The eight platforms are placed uniformly around the central deposit. To indicate the user the next target position, a box with dynamic behavior is placed in a random platform. In this case, the user controls the robotic end-effector to manage a virtual wrench with kinematic behavior in order to pick the target boxes and drop this box in the central deposit. Figure 3 shows a screenshot with the 3D environment of the virtual task with structural information. Basically, the purpose of this task is the same than the 2D task, but with a different visualization level. The workflow to comply the virtual 3D task is:

1. First, the user should approximate the virtual tool to the central deposit to initiate the visualization of perspective target positions.
2. A box appears randomly in any of the eight platforms. The platform is illuminated as visual support, and the virtual wrench is dynamically oriented to the positional target.
3. The user has a limited time to pick the target box. This limited time is shown in a progress bar placed in the up left side of the screen. If the target has not selected, the box disappears and the next target is executed.
4. When the virtual wrench is near to the target, the box is caught. Then the user have to bring the tool to the central deposit to release the box. There is a sound support to indicate that the target is completed.

**Figure 4:**
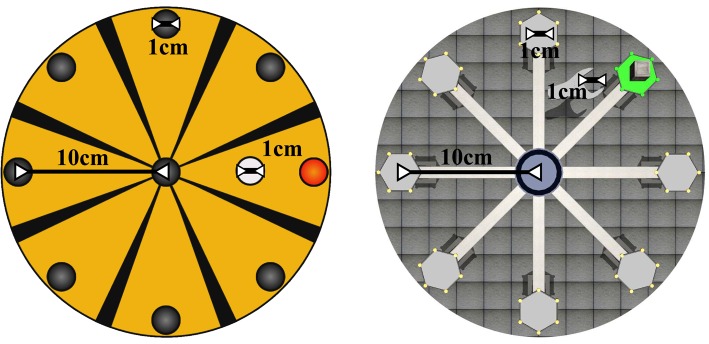
**Structural correlation between 2D Roulette and 3D Roulette (zenithal view)**.

The functionality and structure of the both virtual tasks are the same. This is necessary to an objective comparison of the parameter values obtained by the robotic device. A structural correlation between 2D and 3D tasks is presented in Figure 4. This approach compares the same situation with different external stimulus. The purpose of develop a scenario in three dimensions is provide a correlation more natural between the movement of the robot and the view of the user. In 2D tasks, when the user approaches or moves away the end-effector, the controllable element in the task is moved up or down of the screen. Therefore, many patients tried to force the efector upwards or downwards to put the controllable element in its corresponding place in screen. Associating the horizontal planar movement of the robotic device with the vertical movement in the 2D task displayed in screen, can cause confusion in some patients. In this way, the 3D task replicate in the screen the same type of movement of the robot.

### 2.4. Setup and protocol

Over a 2 months period, the study group has received the therapy treatment assisted by the PUPArm robot with four weekly sessions of 10 min, 36 sessions in total. In the first session, a general evaluation of patient collocation is carried out to get parameters such as the height of the screen or chair, and their mobility range. After, these values are used during the tasks. In this way, the maximum functional range of the specific patient is achieved. Before starting the session, the patient is placed in front of the robotic device in a comfortable position using the parameters obtained in the first evaluation sesion. The monitor that offers the visual feedback is located to 70 cm from the patient. Each session is structured in two blocks of movement training, depending on the virtual task. Between each block, the patients had 3-min rest periods. The session time is organized as follow:

- In the first block of movements, one of the roulette task is choosen randomly. Then, the patient has to perform 32 trials focusing on this selected 3D or 2D task with global movements, both shoulder and elbow. Approximately, this block is completed in 4–5 min.
- Once the 32 trials of the same task have been completed, the patient have a 3-min rest period.
- To finish the session, the second block of movements is performed completing 32 trials with the other roulette task. As with in the first block, the patient have to carry out the same global movements and the time taken to complete these trials are approximately 4–5 min too.

**Figure 5:**
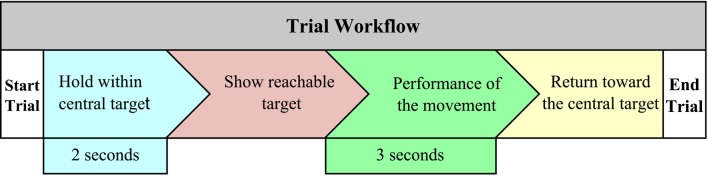
**Workflow to complete one trial**.

The subjects had to reach one of the eight possible targets and return toward the central target in order to complete one trial. Figure 5 shows schematically the workflow to perform one trial. To begin a trial, the subjects had to hold the controllable element through of robotic end-effector for 2 s within the central target. Then, one reachable target was illuminated to indicate the next position where the patient had to place the end-effector. To complete the movement, a limited time of 3 s was given. When the subject reached the target, had to return to the central target without time limit. Therefore, this movement sequence is performed in the 32 planned trials, pointing targets randomly. This protocol was the same to the two virtual tasks, but the elements inside the tasks were different. The following parameters have been calculated from the data recorded by the robotic device. A brief explanation of these parameters (a complete explanation of these parameter can be found in LLinares et al., 2013) are described:

1. Maximum speed: The maximum speed reached by the arm movement.
2. Reaction time: The time elapsed from the indication of the random target and the onset of the arm movement.
3. Path length: The total distance traveled to reach one target.
4. Initial movement: The distance traveled during the initial movement whose trajectory has a deviation with respect the reach target, until this deviation is corrected.
5. Initial movement ratio: Relation between the initial movement and the path length.
6. Initial movement direction error: The angular deviation in degrees, between the optimal path established by the line from the central target to the reach target, and the vector generated by the initial movement.
7. Time: The total time needed to reach one target.
8. Succes rate: The percentage of the trials that have been completed correctly.

Additionally, a study of the system usability has been defined in this work in order to get a quantitative indicator of usability that measures the compliance level front the user expectations, satisfaction level and the performance of the neurorehabilitation system. A type System Usability Scale (SUS) survey Brooke (1996) has been used. A survey has been delivered to each patient after testing the system. This survey had ten questions giving a global view of subjective assessments of usability. Six of these questions had positive character and the remaining questions were negatives to contrast answers. The users had to reply their agree or disagree level regarding the system using a Likert scale (from 1 to 5; Likert, 1974).

## 3. Results

The objective data have been acquired during 36 sessions of the virtual therapy treatment assisted by the PUPArm robot and the main descriptive statistics for each patient have been collected in Table 2. The outcomes are presented in a ten column table to show the kinematics parameter values that assess the quality of patient's motor performance. Each parameter contains two possible values depending on the visualization level of the task provided to the patient. In this case, one value is extracted from 2D Roulette and the other from 3D Roulette to compare the numerical outcomes. The success rate to complete the reaching target can be observed in the last column. The high success rate between 95.10 and 100% indicated that the system was ease of use without complication to perform the virtual tasks. There were no significant differences in the success rate between the two types of visualization.

**Table 2:** **Data acquired by the robotic device**.

**Patient**	**Task**	**Maximum speed**	**Reaction time**	**Path length**	**Initial movement**	**Initial mov. ratio**	**Initial mov. direction error**	**Time**	**Success**
1	2D	104.13	0.69	104.27	72.45	0.70	1.11	7.11	99.61
	3D	115.56	0.89	114.41	83.01	0.74	1.13	8.18	99.61
2	2D	57.07	0.65	114.55	41.60	0.38	3.21	10.35	100
	3D	58.64	0.71	121.53	42.01	0.36	3.17	11.38	98.64
3	2D	91.81	0.85	116.94	58.38	0.52	2.05	11.81	100
	3D	92.58	1.09	119.06	60.60	0.53	1.87	13.59	100
4	2D	118.30	0.71	123.82	69.13	0.61	1.57	13.45	99.67
	3D	134.66	0.89	149.68	78.62	0.60	1.67	16.26	98.58
5	2D	153.19	0.88	197.90	95.59	0.61	1.71	15.41	98.83
	3D	153.40	1.04	250.27	97.45	0.51	2.53	27.04	95.10
6	2D	45.94	0.40	110.07	33.96	0.32	3.56	12.13	98.83
	3D	46.57	0.48	111.14	32.89	0.31	3.77	13.48	97.01
7	2D	63.24	0.64	120.42	42.98	0.37	3.19	16.07	97.13
	3D	60.49	0.77	121.61	41.68	0.36	3.17	15.99	95.53
8	2D	110.09	0.52	105.96	69.78	0.68	1.34	8.03	98.96
	3D	113.72	0.71	115.34	74.28	0.67	1.37	11.76	98.96
9	2D	112.37	0.73	130.40	75.48	0.64	1.73	12.36	100
	3D	121.37	1.05	157.78	83.21	0.61	1.88	13.33	98.27

Measurement units: Maximum Speed, mm/second; Reaction Time, seconds; Path Length, mm; Initial Movement, mm; Initial Movement Ratio, dimensionless; Initial Movement Direction Error, degrees; Time, seconds; Success, %.

A comparative analysis of these parameters has been assessed with both values extracted from 2D and 3D tasks in order to get the patient's performance variation of 3D parameters with respect to 2D parameters. Equation (1) has been used to calculate the percentages of this variation. This equation is applied to each kinematic parameters for all the patients. Therefore, data3D and data2D generalize the numeric value of these kinematic parameters. As each parameter has got associated 2 possible values, the data3D variable records the value of the 3D task, while data2D variable contains the value of the 2D task. The percentages values are gathered in Table 3 including the mean, standard deviation, median and the maximum-minimum value of each parameter of all the data. The positive values indicate the increased percentage that the parameter extracted from the 3D task is higher than the same parameter in 2D task.

(1) $$\begin{array}{l} \left(\frac{data3D-data2D}{data2D}\right)*100 \end{array}$$

**Table 3:** **Variation 3D parameters with respect to 2D parameters in %**.

**Patient**	**Maximum speed**	**Reaction time**	**Path length**	**Initial movement**	**Initial mov. ratio**	**Initial mov. direction error**	**Time**	**Success**
1	10.97	29.27	9.73	14.57	5.79	1.55	15.13	0
2	2.75	9.35	6.09	0.99	−4.69	−2.14	10	−1.36
3	0.83	27.80	1.80	3.80	2.14	−8.99	15.12	0
4	13.83	25.26	20.89	13.72	−3.07	6.74	20.89	−1.09
5	0.13	18.92	26.46	1.94	−17.28	47.90	75.47	−3.78
6	1.36	20.11	0.97	−3.09	−3.79	5.99	11.12	−1.84
7	−4.35	10.99	0.98	−3.03	−3.06	−0.49	−0.47	−1.64
8	3.29	37.77	8.85	6.45	−1.16	2.18	46.58	0
9	8	42.93	20.99	10.24	−4.77	8.99	7.83	−1.73
MEAN	4.09	24.71	10.75	5.07	−3.32	6.86	22.41	−1.27
STD	5.42	10.60	9.14	6.27	5.93	15.37	22.42	1.21
MEDIAN	2.75	25.26	8.85	3.80	−3.07	2.18	15.12	−1.36
MAX	13.83	42.93	26.46	14.57	5.79	47.90	75.47	0
MIN	−4.35	9.35	0.97	−3.09	−17.28	−8.99	−0.47	−3.78

**Figure 6:**
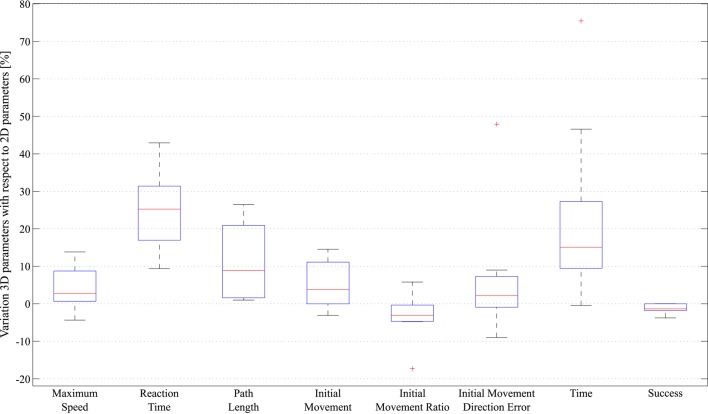
**Statistical analysis of the data acquired by the robotic device, represented in box plots**.

These parameters are plotted as box plots in Figure 6 to provide a general vision of the data distribution. On a box plot, the boxes are divided by a horizontal segment that indicates the position of the median value. Therefore, the relation between this value and the 25th and 75th percentiles, represented by the bottom and top of the box, can be observed. The boxes are located on a segment whose extremes the minimum and maximum values of the parameter. In this box plots, the outliers values have been marked with the “+” symbol and an asymmetric distribution appears in the boxes.

**Figure 7:**
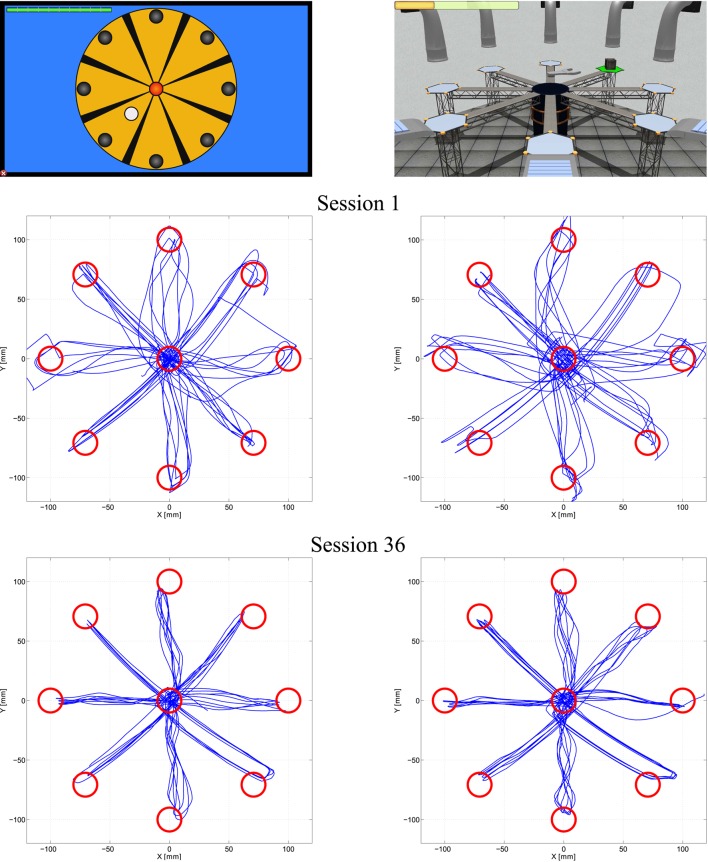
**Movement trajectories to reach the targets by one patient**. Sensorimotor function assessment for two tasks. In the left are shown trajectories performed in 2D task during the first and the last session. In the right side are shown the trajectories performed in 3D task.

To evaluate the sensorimotor function after the virtual therapy based in robotic-assisted neurorehabilitation, the movement trajectories performed by one subject in the first and the last session are shown in Figure 7. The left side of this figure corresponds to trajectories during the visualization of the 2D Roulette, while the right side was carried out with the 3D Roulette. In both tasks, they were more erratic trajectories in the first session when the subject attempted to reach the reachable targets. However, the patients performed more correct trajectories when they used the task with 2D environment. The trajectories presented a more irregular paths in 3D task. Furthermore, less deviations of trajectory are performed when the patients had to reach the central target in the 2D task. Regarding the first session, in the 3D Roulette the trajectories between the reached target and the central target presented a more length than the 2D task and the deviation error and the time to reach the targets is higher (see Table 3 and Figure 7). Therefore, the patients present more difficults to achieve the targets in the 3D task when they started the therapy, using the proposal system. This may mean that the usage of tasks displayed with 2D graphics, hence with less level of detail, is easier to perceive and is better adapted to users who have not used this type of system. In Figure 7 an improvement in the control of the system by the patient can be observed. This fact suggests that the sensorimotor performance of the patient is increased due to the repetition of the movements with the arm limb for practice motor skills.

A statistical analysis of these quantitative elements has been performed to verify the bivariate correlation of the kinematic and kinetic parameters between them, in order to find out the level and direction of the correlation. In this way, the type of visualization that generate better relations between kinematic parameters can be analyzed, and consequently, a better assessment of the results can be achieved. This analysis is performed from calculating the Pearson correlation coefficient and the level of significance to indicate if there is a correlation between each pair of the study parameters. Table 4 shows the correlation matrix with the Pearson correlation coefficient and the level of significance of each pair of parameters assigned to record the data obtained in the 2D visualization. Meanwhile, Table 5 records the correlation matrix with the data generated by the 3D task.

**Table 4:** **Correlation matrix of each pair of parameters assigned for the data obtained in the 2D visualization**.

		**Maximum speed**	**Reaction time**	**Path length**	**Initial movement**	**Initial mov. direction error**	**Time**
Maximum	R	1	0.645	0.654	0.986^**^	−0.842^**^	0.075
speed	Sig.	-	0.060	0.056	0.000	0.004	0.848
Reaction	R	0.645	1	0.605	0.640	−0.457	0.317
time	Sig.	0.060	-	0.084	0.063	0.217	0.406
Path	R	0.654	0.605	1	0.642	−0.158	0.614
length	Sig.	0.056	0.084	-	0.062	0.685	0.078
Initial	R	0.986^**^	0.640	0.642	1	−0.852^**^	0.003
movement	Sig.	0.000	0.063	0.062	-	0.004	0.994
Initial Mov.	R	−0.842^**^	−0.457	−0.158	−0.852^**^	1	0.386
direction error	Sig.	0.004	0.217	0.685	0.004	-	0.305
Time	R	0.075	0.317	0.614	0.003	0.386	1
	Sig.	0.848	0.406	0.078	0.994	0.305	-

R, Pearson correlation; Sig, Level of significance;

** The correlation is significative in the level 0.01.

**Table 5:** **Correlation matrix of each pair of parameters assigned for the data obtained in the 3D visualization**.

		**Maximum speed**	**Reaction time**	**Path length**	**Initial movement**	**Initial mov. direction error**	**Time**
Maximum	R	1	0.729^*^	0.684^*^	0.982^**^	−0.713^*^	0.445
speed	Sig.	-	0.026	0.042	0.000	0.031	0.230
Reaction	R	0.729^*^	1	0.530	0.745^*^	−0.594	0.321
time	Sig.	0.026	-	0.142	0.021	0.092	0.400
Path	R	0.684^*^	0.530	1	0.649	−0.002	0.899^**^
length	Sig.	0.042	0.142	-	0.059	0.996	0.001
Initial	R	0.982^**^	0.745^*^	0.649	1	−0.746^*^	0.360
movement	Sig.	0.000	0.021	0.059	-	0.021	0.342
Initial mov.	R	−0.713^*^	−0.594	−0.002	−0.746^*^	1	0.253
direction error	Sig.	0.031	0.092	0.996	0.021	-	0.512
Time	R	0.445	0.321	0.899^**^	0.360	0.253	1
	Sig.	0.230	0.400	0.001	0.342	0.512	-

R, Pearson correlation; Sig, Level of significance.

* The correlation is significative in the level 0.05.

** The correlation is significative in the level 0.01.

**Figure 8:**
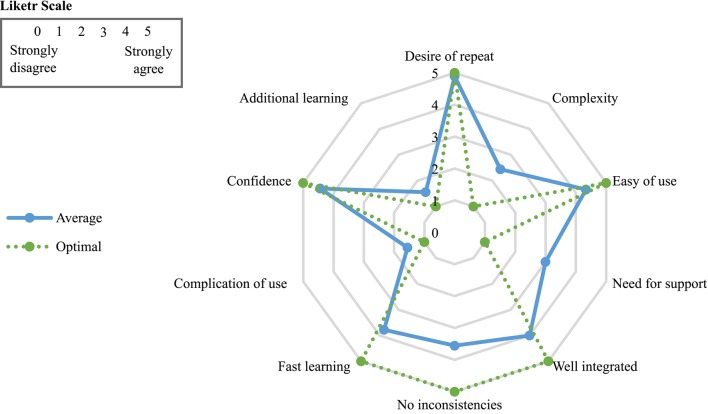
**Average of the survey patient responses**.

A usability measure of the neurorehabilitation system has been calculated when all patients have completed their corresponding SUS surveys. Table 6 shows the statements that cover a variety of aspects of system usability, such as need for support, training and complexity. Also, the table gather the outcomes provided by each user on a 5 point scale ranging from “strongly agree” to “strongly disagree.” The average of the patient assessment of the aspects dealt in the survey are collected in Figure 8. In this radial graph, the pointed line represents the optimal response for getting the highest subjective level of usability. The continued line is the response average of the nine patients. Generally, the response of the patients was widely similar to the optimal responses, except for the question about the needed for technical support. This mean a high usability rate.

**Table 6:** **Questions of the survey and the patient's answers**.

		**N Patient**								
**N**	**Question**	**1**	**2**	**3**	**4**	**5**	**6**	**7**	**8**	**9**
1	I think that I would like to use this system frequently	5	5	5	4	5	5	5	5	5
2	I found the system unnecessarily complex	5	5	1	2	1	3	3	1	1
3	I thought the system was easy to use	5	5	5	4	4	3	3	5	5
4	I think that I would need the support of a technical person to be able to use this system	3	3	2	2	1	5	4	3	4
5	I found the various functions in this system were well integrated	4	4	5	4	5	3	3	4	4
6	I thought there was not any inconsistency in this system	5	5	1	2	5	3	5	5	1
7	I would imagine that most people would learn to use this system very quickly	4	4	5	4	5	3	4	3	2
8	I found the system very cumbersome to use	1	1	1	1	1	3	4	1	1
9	I felt very confident using the system	5	5	5	4	5	3	3	5	5
10	I needed to learn a lot of things before I could get going with this system	1	1	1	1	1	3	4	1	1

**Figure 9:**
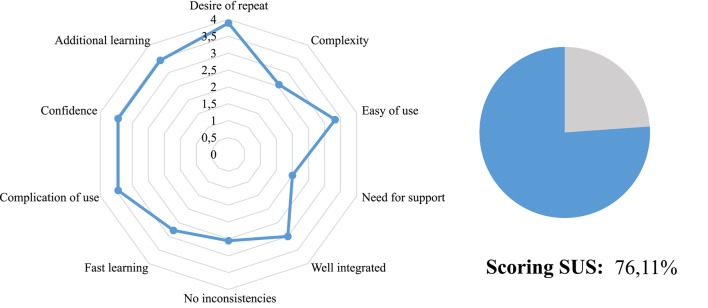
**Score contribution from each aspect of the survey**.

For getting an usability interpretation or punctuation represented by a percentage indicator, the numerical contribution of each point are added depending on the question. Each item's score contribution has a range from 0 to 4, as can be observed in Figure 9. Then, the result of the sum are multiplied by 2.5 to get the SUS scoring (Brooke, 1996). The total SUS score of the survey was 76.11%. It is a very positive indicator that reflects a high satisfaction and engagement of the assessed patients. A percentage more high means a better usability level.

## 4. Discussion

The influence of applying 2D or 3D therapeutic games in the performance of upper limb rehabilitation in post-stroke patients has been presented in this study. Currently, this type of study have not been addressed yet in the scientific literature or the issues dealt in this field are discussed by subjective assessments. For these reason, a quantified correlation between upper-limb motor function and the visualization of the reaching task was assessed in an objective way computing kinematic parameters provided by PUPArm robotic device. At first glance, the analysis of the kinematic parameters with two types of visualization has provided very similar results comparing nominal values. However, some little differences in sensorimotor performance have been found depending upon the visualization of the task based on peripheral or perspective targets, viewing these values and performing an analysis of the correlation between kinematic parameters. Each patient achieved similar outcomes when performing both task with 2D and 3D environments in all sessions during the therapy. However, some patients obtained better results than others showing a variation in the sensorimotor abilities. These changes may be due to age, motor damage or level of cerebral injury that affect to the efficiency of cognitive and physiological processes.

**Figure 10:**
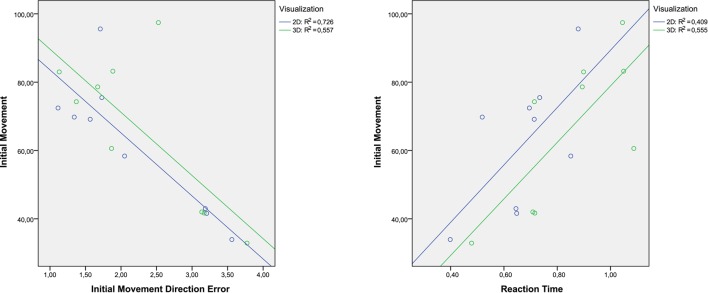
**Dispersion diagram with the most significant variables that affect the initial deviation of the trajectories**.

Perform straight trajectories to reach the targets was an important purpose of the tasks. In the first sessions, the most of the targets in the both tasks were achieved with erratic trajectories. For same peripherals and perspective targets, the trajectories presented different deviations in each trial. This fact happened for all patients. However, as is shown in Figure 7, the reachable targets that required diagonal trajectories were reached with better kinematic movements in the 2D task. More rectilinear trajectories were performed. In the 3D tasks, deviant trajectories are observed in all targets. Regarding the central target, the trajectories were more precise with less deviation in the 2D task. The left graph in the Figure 10 shows the dispersion diagram between the Initial Movement and the Initial Movement Direction Error. These two parameters directly affect the correct performing of the trajectories and were highly significative in the correlation analysis in both visualization types. This correlation was negative and the lineal asociation degree was stronger in the 2D task. This means that the deviation error when the patient start the arm movement is corrected with less initial distance to reach the better direction trajectory to the target with 2D environments. This fact, is because the zenithal view allows to visualize better the path traveled, facilitating to carry out more straight trajectories.

Consequently, the best trajectories performance in the 2D tasks may suggest that the 2D environments are more appropiate scenarios to generate tasks that provide a better sensorimotor control when the patients start to use the system. For these reason: the trajectories were straighter and had less deviations. In the last session, the movement trajectories were corrected significantly to the point to achieve paths almost without deviation. While the targets are reached satisfactorily with stable trajectories. Comparing the outcomes of the first and the last session can be observed that the trajectories improve with the patient's experience. This enhancement indicates a positive assessment in the recovery of the sensorimotor function of the patients, as is happened in LLinares et al. (2013), Badesa et al. (2014b). Generally, the success rates in all session during both tasks were quite high. The targets were achieved practically in all trials by the patients. The high values of the success rate insinuate that the tasks were not complex and the targets were recognized clearly.

On the other hand, differences between tasks in terms of kinematic and kinetic parameters can be found in Tables 2, 3 and Figure 6. For all patients, the time reaction in 3D task was higher than 2D task, which implies that the increasing of the immersion level in the environment provokes unnecessary distractions to the patients. Thus, the patient's concentration level is augmented with less detail level in the virtual environment. Nevertheless, the correlation between the Initial Movement and the Reaction Time (right graph in Figure 10) is more significative in the 3D task, indicating that the reaction time in 2D task produces the need to perform a longer correction distance to compensate the deviation error, although the nominal value of reaction time in 2D tasks is smaller than in 3D tasks. This, also affects in the trajectory performing.

**Figure 11:**
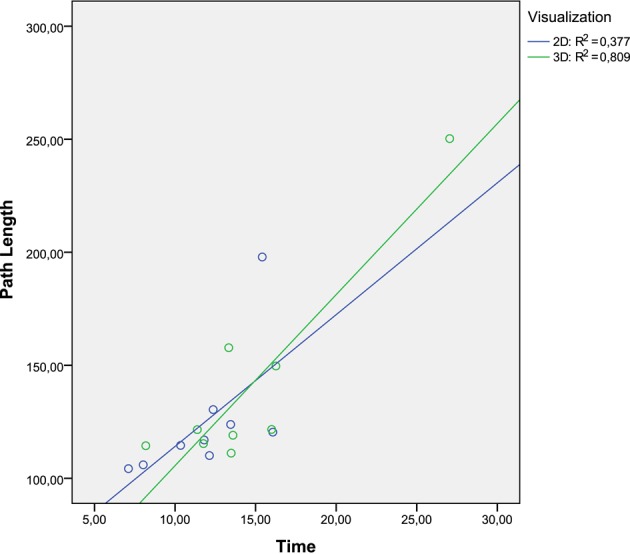
**Dispersion diagram between Path Length and Time parameters**.

While conducting trajectories to reach the targets, the robot manipulator was displaced to a lesser extent in the 2D task. Therefore, the path length was higher in the 3D task for all the patients. Consequently, the total time to complete all the trials was upper in the 3D task. Only one patient has required less time to perform the 3D task. It may be that the patients guide the robotic manipulator better when they are observing a 2D environment. In the 3D task, the depth of the scenario increases the difficulty level to complete the targets. The patients have adapted better to the 2D task. Although the path length was higher in the 3D task, the times to complete both tasks do not differ substantially due to the patients achieved a maximum speed of movement in the 3D task. The correlation between the Length Path and the total time to reach one target, only the 3D task has a relevant level of significance with a very strong relation value (Figure 11). The 3D visualization provides a linear behavior between these two parameters and consequently allow to perform more natural movements, due to a better association between the movement of the end-efector and the avatar movement in the virtual task.

All trials can be performed in an optimal way following a straight trajectory from the central target to the reachable target and vice versa. However, a deviation has been produced when the patients started the movement to complete all trials. This deviation entails that the patients accomplished an incorrect path before the trajectory direction was corrected to reach the optimal path. This situation is more accentuated in the 3D task in almost all patients, as it be observed through the nominal analysis of the Initial Movement and Initial Movement Direction Error, and the dispersion diagram shown in Figure 10.

**Figure 12:**
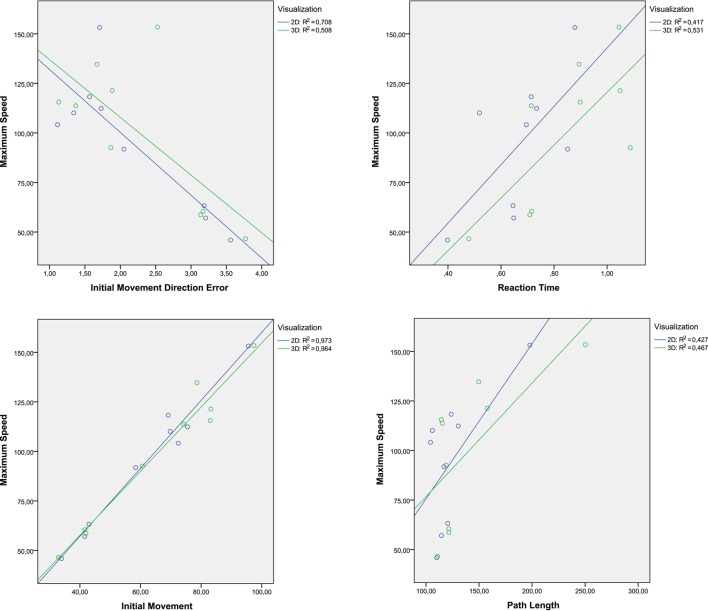
**Dispersion diagrams with the significative correlation of speed maximum**.

Regarding the Maximum Speed, there are strong coefficients of correlation between it and the others parameters. Figure 12 collects 4 dispersion diagrams where the Maximum Speed is analyzed with the parameters that provide a higher correlation. The two graphs placed in the left side indicate that the Maximum Speed has better correlation with the Initial Movement and the Initial Movement Direction Error in the 2D task. In general it can be said that the 2D task provides a better control of the end-effector velocity, taking into account the start of movement. The correlation between the Maximum Speed and Reaction Time or Path Length was better in the 3D task. These two correlations may suggest that the 3D task allows the user to perform longer trajectories but with more natural and dynamic movements. With the rest of parameters, correlation significative was not found.

In conclusion, the main purpose of this study was to verify if there were differences in patterns of kinematic movements of post-stroke patients assisted by a robotic device when environments were visualized in two and three dimensions. Despite the similarity in the results and the correlation analysis, the hypothesis that consists of showing a visualization environment more natural increasing the immersion level did not provide many improvements with regard to an environment simpler. Therefore, the using of 2D environments in virtual therapy may be a more appropriate and comfortable way to perform tasks for upper limb rehabilitation of post-stroke patients, in terms of accuracy in order to effectuate optimal kinematic trajectories. Knowing what virtual environment is more appropriate to each user, therapies with better assessment tools can be implemented and adapted to the patient's needs and limitations (Morales et al., 2014). Depending of the assessment objective about the sensorimotor function such as time reaction, speed or stability of movement, one type of visualization or other can be used. This is highly advantageous in the clinical environment to enhance the course of the rehabilitation of sensorimotor function and reduce the recuperation times.

## Author contributions

Conceived and designed the experiments: FB, NG, JS. Performed the experiments: LL, SE, JD. Collected the data and processing them: LL, AB, SE. Analyzed the data and interpreted the results: LL, FB, JS, NG. Wrote the paper: LL, JD, FB, NG. All authors read and approved the final manuscript.

### Conflict of interest statement

The authors declare that the research was conducted in the absence of any commercial or financial relationships that could be construed as a potential conflict of interest.
